# Prospective CERAD Neuropsychological Assessment in Patients With Multiple System Atrophy

**DOI:** 10.3389/fneur.2022.881369

**Published:** 2022-07-19

**Authors:** Fabian Maass, Peter Hermann, Daniela Varges, Sabine Nuhn, Christoph van Riesen, Ala Jamous, Niels K. Focke, Manuel Hewitt, Andreas Leha, Mathias Bähr, Inga Zerr

**Affiliations:** ^1^Department of Neurology, University Medical Center Göttingen, Göttingen, Germany; ^2^German Center for Neurodegenerative Diseases (DZNE), Göttingen, Germany; ^3^Department of Neuroradiology, University Medical Center Göttingen, Göttingen, Germany; ^4^Department of Medical Statistics, University Medical Center, Göttingen, Germany; ^5^Cluster of Excellence Nanoscale Microscopy and Molecular Physiology of the Brain (CNMPB), University Medical Center Göttingen, Göttingen, Germany

**Keywords:** multiple system atrophy (MSA), neuropsychology, CERAD neuropsychological assessment battery, trail making test (TMT), executive dysfunction

## Abstract

The objective of the study was to characterize the pattern of cognitive dysfunction in patients with multiple system atrophy (MSA) applying a standardized neuropsychological assessment. A total of 20 patients with the diagnosis of probable or possible MSA were enrolled for neuropsychological assessment applying the CERAD plus battery. All patients were tested at baseline and 14/20 patients received additional follow-up assessments (median follow-up of 24 months). Additionally, relationship between cortical thickness values/subcortical gray matter volumes and CERAD subitems was evaluated at baseline in a subgroup of 13/20 patients. Trail Making Test (TMT) was the most sensitive CERAD item at baseline with abnormal performance (z-score < −1.28) in one or both pathological TMT items (TMT-A, TMT-B) in 60% of patients with MSA. Additionally, there was a significant inverse correlation between the volume of the left and the right accumbens area and the TMT A item after adjusting for age (left side: *p* = 0.0009; right side *p* = 0.003). Comparing both subtypes, patients with MSA-C had significant lower values in phonemic verbal fluency (*p* = 0.04) and a trend for lower values in semantic verbal fluency (*p* = 0.06) compared to MSA-P. Additionally, patients with MSA-C showed significantly worse performance in the TMT-B task (*p* = 0.04) and a trend for worse performance in the TMT-A task (*p* = 0.06). Concerning longitudinal follow-up, a significant worsening in the TMT-B (*p* = 0.03) can be reported in MSA. In conclusion, frontal-executive dysfunction presents the hallmark of cognitive impairment in MSA.

## Introduction

Multiple system atrophy (MSA) is a rare sporadic neurodegenerative disease mainly characterized by autonomic dysfunction in combination with cerebellar symptoms or parkinsonism. A total of two different motor subtypes are commonly differentiated: the cerebellar (olivopontocerebellar atrophy; MSA-C) and the parkinsonian subtype (striatonigral degeneration, MSA-P) (1).

Besides prominent motor symptoms, different non-motor symptoms can also be found in MSA (2). According to current diagnostic criteria, the development of severe dementia is defined as a feature not supporting the diagnosis of MSA but cognitive impairment ranging from single to multi-domain mild cognitive impairment (MCI) is now accepted as a common feature (3, 4). Recently, Koga et al. analyzed the medical history of 171 patients with autopsy confirmed MSA from the Mayo Clinic Brain Bank and cognitive impairment could be found in 35% of cases (5).

Pathologically, patients with cognitive impaired MSA have been described to show more neuronal cytoplasmic inclusions in the limbic regions (6). On the other side, occurrence of Alzheimer's disease-related changes in the presence of alpha synuclein pathology might also contribute to cognitive dysfunction (7).

The characteristics of MCI in MSA have been described to be similar to patients with non-demented Parkinson's disease, showing prominent executive dysfunction and visuospatial disturbances. Due to limited sample sizes and heterogenous cohorts, only conflicting results concerning the differences in the pattern of cognitive impairment between the MSA-C and MSA-P subtypes have been reported (4).

More importantly, due to a limited number of longitudinal trials on cognition in MSA, evolution of cognitive impairment over time is still not well characterized, and further studies are needed for clarification (4). Previous longitudinal trials reported different results ranging from stable cognitive function to significant worsening of multiple cognitive domains with emphasis of frontal-executive functioning, mainly applying one follow-up examination and time periods between 12 and 21 months (8–13). Interpretation is especially limited by heterogenous test assessments and the lack of longer time periods and/or multiple time points of testing.

Here, we present a monocentric prospective trial on the longitudinal evolution of cognitive deficits in patients with MSA-P and MSA-C applying the standardized Consortium to Establish a Registry for Alzheimer's Disease-Neuropsychological Assessment Battery (CERAD-NAB) and multiple time points of follow-up (median follow-up 24 months, 25p−75p:13–45 months). The CERAD battery has been intensively used in the field of Alzheimer's dementia and allows standardized evaluation of cognitive dysfunction also implementing age, gender, and education adjustments based on big norm populations. Additionally, the CERAD plus version–as applied for this study–also includes the items on processing speed and frontal- executive functioning (14), allowing to assess typical domains as involved in MSA (4).

Additionally, widespread cortical and subcortical brain atrophy has been described in patients with MSA, potentially linked to cognitive dysfunction (4). Therefore, we also evaluated potential correlations between CERAD subitems and the cortical thickness/subcortical volumes at baseline, as quantified by advanced MRI modalities (subcortical gray matter volumetry, cortical thickness analysis).

## Methods

### Subjects

Patients with the diagnosis of probable or possible multiple system atrophy according Gilman criteria (3) were enrolled in the Dept. of Neurology, University Medical Center, Göttingen, Germany between 2012 and 2019. Patients were eligible independent of disease duration, disease severity, or clinical subtype (MSA-P/MSA-C). Neurological examination, baseline neuropsychological assessment, and routine MRI imaging were performed in the context of the clinical routine. If feasible, patients received follow-up examinations prospectively for longitudinal neuropsychological assessment. The Unified Parkinson's Disease Rating Scale (UPDRS) part III was used for motor assessment (15). Disease duration was defined as the time span since the awareness of the first motor (parkinsonian or cerebellar) symptoms. Permissions of the local ethics committees have been obtained (Ethics Committee of the University Medical Center Göttingen 19/11/09 and 01/09/21) and written consent was provided by all patients. The study conforms with the Code of Ethics of the World Medical Association (Declaration of Helsinki).

### Neuropsychological Assessment

All baseline and follow-up neuropsychological assessments were performed applying the German plus version of the “Consortium to Establish a Registry for Alzheimer's Disease Neuropsychological Assessment Battery” (CERAD-NAB) (14, 16). First two follow-up examinations were performed in the course of a half-yearly period, and further follow-up examinations were performed yearly. The standard version contains tasks for the evaluation of verbal fluency (animal naming), a modified version of the Boston Naming Test, global cognition (Mini-Mental State Examination–MMSE), verbal memory (word list learning, delayed recall), and constructional practice and delayed recall (17). The plus version further contains three additional items on processing speed (Trail Making Test A–TMT A) and executive/frontal functioning (Trail Making Test B–TMT B, letter fluency: S-words) (14). The Trail Making Test B/A ratio (TMT B/A) was calculated as an additional measure of executive function (18). Age-, gender-, and education-adjusted results according to the CERAD-NAB of a German-speaking norm population were used, and a z-score < −1.28 (10 percentile threshold) was considered as a significant neuropsychological dysfunction (19).

### MRI Analysis

Routine MRI was obtained from either a 1.5T Siemens Magnetom Avanto, a 3T Siemens Magnetom Prisma fit or a 3T Siemens Magnetom Trio Tim system. Only datasets containing a 3D-T1-weighted magnetization-prepared rapid gradient-echo sequence (MPRAGE) and without relevant artifacts, e.g., from motion, were used for further image post-processing. Cortical thickness analysis was performed based on surface-based pipeline using Freesurfer v6.0.0 (20, 21). From this processing, gray matter cortical thickness (in mm) was used for the further analysis using the Desikan-Killiany cortical parcellation scheme (22). Additionally, volumes of deep gray matter structures were calculated using the Freesurfer reconstructed images (ASeg stats). Regional subcortical volumes were normalized by the total intracranial volume and used for further analysis. Total brain volume measurements, as well as gray and white matter measurements, have been described to be robust across MRI field strength for Freesurfer (23). Detailed information on the Freesurfer image analysis suite and associated methods can be found on http://surfer.nmr.mgh.harvard.edu/.

### Statistical Analysis

Data distribution was assessed visually (quantile–quantile plot) and by applying Shapiro–Wilk normality test. Longitudinal changes in CERAD subitems over time were evaluated fitting a mixed model as implemented in GraphPad Prism. This mixed model uses a compound symmetry covariance matrix and is fit using restricted maximum likelihood (REML). Uncorrected Fisher's least significant difference test was used as follow-up test. Differences between the MSA-P and the MSA-C subgroups at baseline were evaluated applying Mann–Whitney U test. Qualitative data were compared using chi-squared test. Correlation analysis between two variables was performed using Spearman's rho and adjusted for age applying multiple linear regression. Correlation or regression analysis was adjusted for multiple comparisons according to Bonferroni and additionally by usage of a less restrictive unadjusted alpha of *p* ≤ 0.01. All analyses were performed using GraphPad Prism 9.0.0.

## Results

### Participant Characteristics

A total of 20 patients with MSA were enrolled for this study. Baseline clinical characteristics are summarized in Table 1. A total of six patients received baseline assessment but refused further longitudinal assessment or dropped out due to progressive disability. A total of 14 patients received additional follow-up assessments (Table 2). Median follow-up was 24 months (25–75p:13–45 months).

**Table 1:** Characteristics of the study population at baseline.

**Clinical characteristics**	**Overall**	**MSA-C**	**MSA-P**	***p-*value**
Number of patients Probable / possible MSA	20 13/7	8 5/3	12 8/4	ns
Age, years	68 (62–73)	66 (62–72)	70 (62–74)	ns
Gender (male / female)	11/9	4/4	7/5	ns
Disease duration, years	3 (2–5)	3 (2–4)	3 (1–7)	ns
UPDRS III score Follow-up, months Education, years Urinary incontinence (yes / no) Orthostatic hypotension (yes / no)	28 (20–57) 24 (13–45) 13 (12–17) 16/4 12/8	20 (9–47) 15 (12–48) 13 (11–17) 6/2 4/4	35 (21–59) 24 (17–43) 14 (12–18) 10/2 8/4	ns ns ns ns ns

*MSA-C, Multiple system atrophy–cerebellar subtype; MSA-P, Multiple system atrophy–parkinsonian subtype; UPDRS, Unified Parkinson's Disease Rating Scale. Data are presented as median (25–75p). ns, not significant*.

**Table 2:** Summary of the CERAD neuropsychological plus battery subitem results over time.

	**Baseline (median duration)**	**Visit 2 6 months**	**Visit 3 12.5 months**	**Visit 4 24 months**	**Visit 5 36 months**	**Visit 6 47 months**	***p*-Value**
Verbal fluency (animals)	17 (25); *n* = 20	13 (24); *n* = 9	16 (23); *n* = 12	23 (23); *n* = 7	20 (9); *n* = 2	17 (18); *n* = 5	0.58
Boston naming test	15 (4); *n* = 20	15 (5); *n* = 8	14 (5); *n* = 11	14 (2); *n* = 7	14 (2); *n* = 2	14 (2); *n* = 5	0.54
MMSE total score	28 (7); *n* = 20	28 (6); *n* = 11	28 (6); *n* = 12	29 (2); *n* = 7	26 (5); *n* = 3	27 (7); *n* = 5	0.23
Word list learning sum	20 (15); *n* = 19	21 (11); *n* = 7	20 (18); *n* = 11	25 (10); *n* = 7	25 (2); *n* = 2	21 (9); *n* = 5	0.52
Word list recall	7 (7); *n* = 20	8 (4); *n* = 6	7 (10); *n* = 11	9 (5); *n* = 7	9 (0); *n* = 2	7 (4); *n* = 5	0.32
Constructional praxis	10 (6); *n* = 20	10 (4); *n* = 9	10 (3); *n* = 11	10 (2); *n* = 7	11 (1); *n* = 2	9 (4); *n* = 5	0.52
Constructional praxis recall	8 (7); *n* = 19	8 (11); *n* = 9	10 (6); *n* = 11	8 (5); *n* = 7	6 (3); *n* = 2	8 (4); *n* = 4	0.09
Trail making test A	56 (97); *n* = 19	49 (74); *n* = 9	65 (134); *n* = 11	55 (63); *n* = 7	65 (28); *n* = 2	70 (104); *n* = 5	0.18
Trail making test B	135 (265); *n* = 18	147 (230); *n* = 8	116 (254); *n* = 10	118 (178); *n* = 7	274 (411); *n* = 2	131 (226); *n* = 3	0.03*
Trail making test B/A	2.9 (3.3); *n* = 18	2.2 (4.0); *n* = 8	2.5 (2.3); *n* = 10	2.2 (1.8); *n* = 7	3.7 (4.7); *n* = 2	2.0 (2.4); *n* = 3	0.65
Verbal fluency (s-words)	9 (14); *n* = 20	10 (14); *n* = 9	11 (15); *n* = 12	12 (16); *n* = 7	10 (9); *n* = 2	15 (14); *n* = 5	0.51

A significant worsening of the Trail Making Test B (TMT-B) (p=0.03*) could be demonstrated applying a mixed model based on restricted maximum likelihood. Data are presented as median (range = max.-min.) sample size. Median.

### Baseline Cognitive Assessment (CERAD Plus Battery)

**Figure 1:**
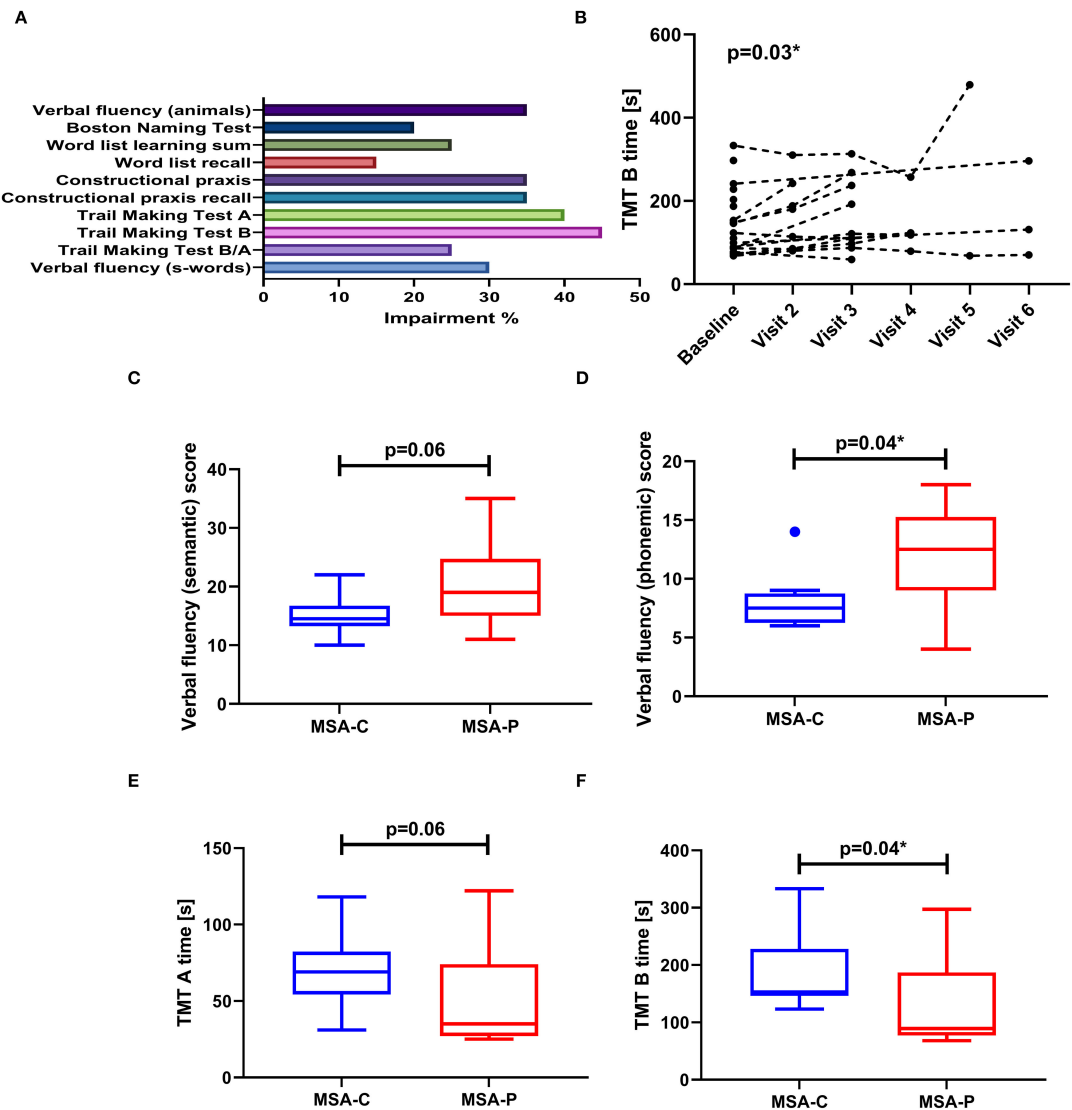
**(A)** Bar graph showing the proportion of impairment (z-score < − 1.28) considering the CERAD battery plus subitems at baseline examination (MSA *n* = 20). **(B)** Line plot demonstrating significant worsening of the Trail Making Test B (TMT-B) subitem over time (*p* = 0.03*), reflected by an increase in time needed to complete this task. **(C–F)** Comparison of MSA-C and MSA-P motor subtypes regarding baseline cognitive function. Data are presented as boxplot (with median, IQR and outlier according to Tukey's rule).

Median MMSE total score for the recent cohort was 28 (25–75p: 27–29) points. The proportion of pathological CERAD plus subitems according to age-, gender-, and education-adjusted z-scores is summarized in Figure 1A. Impairment (z-score < −1.28) can be demonstrated basically in all tested domains to a different degree (language functions, memory functions, visuospatial functions, psychomotor speed, and executive functions, Figure 1A). Percentage of an impaired item considers the whole cohort: verbal fluency (animal naming) 35%, Boston Naming Test 20%, word list learning 25%, word list recall 15%, constructional praxis 35%, constructional praxis recall 35%, and letter fluency (s-words) 30%. TMT was quite sensitive for cognitive dysfunction in MSA, yielding one or both pathological TMT items (TMT-A 40%, TMT-B 45%) in 60% of patients with MSA. A pathological TMT B/A ratio was found in 25% of cases. There was no correlation between the UPDRS part III motor score and the TMT A or TMT B item (*p* > 0.05).

At baseline, 12 patients presented with a multiple domain MCI. A total of six patients showed a single domain MCI of which two converted into a multiple domain MCI and one into a dementia over time. Then, one patient showed no cognitive impairment but converted into single domain MCI and one patient showed a beginning dementia at baseline.

Comparing both subtypes, patients with MSA-C presented with poor language function reflected by significant lower values in phonemic verbal fluency (*p* = 0.04) and by a trend for lower values in semantic verbal fluency (*p* = 0.06) (Figures 1C,D). Additionally, patients with MSA-C also showed poor performance in the TMT-B task (*p* = 0.04) and a trend for poor performance regarding the TMT-A task (*p* = 0.06). There were no differences neither in age nor in disease duration between both groups (*p* > 0.05), so no adjustment had to be performed.

### Longitudinal Cognitive Assessment (CERAD Plus Battery)

Follow-up assessment is summarized in Table 2. A mixed effect model was applied to evaluate longitudinal changes in CERAD subitems. Over time, there were no significant changes in verbal fluency (semantic), naming, global cognition (MMSE), word list learning, word list recall, constructional praxis, constructional praxis recall, and verbal fluency (phonemic), respectively (*p* > 0.05). There was a borderline significant worsening in TMT B (*p* = 0.03; Figure 1B), but not in TMT A or TMT B/A (*p* > 0.05). Follow-up testing revealed main differences in TMT B between baseline and visit 3 (*p* = 0.03), baseline and visit 5 (*p* = 0.002), visit 2 and visit 5 (*p* = 0.01), visit 3 and visit 5 (*p* = 0.04), and between visit 4 and visit 5 (*p* = 0.01).

### Correlation Between Baseline Cognitive Parameter and Cortical Thickness Analysis

Correlation analysis was performed between the baseline CERAD battery plus subitems and the cortical thickness values, as well as the subcortical gray matter volumes. Multiple linear regression was applied to adjust for age as a potential confounder. Baseline MRI data were available for 13/20 patients.

Concerning cortical thickness, no significant correlations could be demonstrated if corrected for multiple testing according to the Bonferroni method (adjusted alpha *p* ≤ 0.0001). Applying a less restrictive unadjusted alpha of *p* ≤ 0.01, a significant correlation between the word list learning item and left superior temporal gyrus thickness (*p* = 0.001, r = 0.82) could be demonstrated, even if adjusted for age (*p* = 0.004).

Concerning subcortical gray matter structures, significant correlation between the volume of the left and the right accumbens area and the TMT A item (left side: *p* = 0.004, r = −0.78; right side: *p* = 0.007, r = −0.74) could be demonstrated, still being significant after adjusting for age (left side: *p* = 0.0009; right side *p* = 0.003).

## Discussion

Here, we present a study on cognitive impairment in patients with multiple system atrophy applying a comprehensive standardized test assessment (CERAD-NAB plus version). We report an involvement of multiple cognitive domains at baseline and a worsening of the Trail Making Test B item over time, potentially reflecting progressive frontal-executive dysfunction.

The pattern of cognitive impairment in MSA was analyzed cross-sectionally at baseline in all patients and additionally at follow-up investigation in 14/20 patients. Median MMSE total score for the current cohort was 28 points as an indicator for relatively preserved global cognitive function. This is in accordance with previous studies on cognition in MSA (10, 12, 24). Indeed, severe dementia has been only reported in 12% of MSA cases in a study applying the Movement Disorder Society Parkinson's disease dementia criteria (25).

On the other hand, impairment (z-score < − 1.28) in at least one cognitive domain could be demonstrated in 19/20 patients. Impairments were found in all tested domains to a different degree (language functions, memory functions, visuospatial functions, processing speed, and frontal-executive functions) (Figure 1A). The TMT items as the indicators for processing speed and executive function revealed the most frequent abnormalities with one or both pathological TMT items (TMT-A and TMT-B) in 60% of patients with MSA. Concerning potential influences by motor disability, correlation analysis between the UPRDRS motor score and TMT performance showed no significant relationship. Additionally, the TMT B/A ratio was calculated which has been discussed to present a more stable item regarding executive function in the presence of motor dysfunction (18). Here, 25% of patients showed a pathological index pointing to impaired executive functioning. Verbal fluency (as another indicator of executive functioning) and constructional praxis as indicators for visuospatial functioning were also quite often impaired, revealing pathological results in >30% of our cohort.

This is in high accordance with typical pattern of cognitive impairment in MSA as described in the literature. As reviewed by the Neuropsychology Task Force of the Movement Disorder Society (MoDiMSA), frontal-executive dysfunction presents the most prominent cognitive disturbance in MSA, affecting up to 49% of patients. Patients with MSA have also been reported to experience visuospatial and constructional difficulties in multiple studies but with less evidence compared to executive function due to inconsistent reports (4).

Cortical and subcortical atrophy pattern in MSA has been described to correlate with impairment in different cognitive domains (4). Therefore, we evaluated potential correlations between CERAD subitems and the cortical thickness values/subcortical volumes at baseline. Interestingly, an inverse correlation between the baseline TMT A item and the volume of the left and also the right accumbens nucleus could be demonstrated, respectively. Atrophy of the accumbens nucleus–potentially hinting to an involvement of the dopaminergic mesolimbic system–has been prior reported in idiopathic Parkinson's disease (PD) and also in patients with MSA (26). In PD, dopa-resistant apathy has been reported to correlate with the severity of accumbens atrophy (27), probably affecting processing speed as measured by TMT-A.

Comparing the two motor subtypes, patients with MSA-C in our cohort presented with poor performance in language items compared to MSA-P. This was reflected by significant lower values in phonemic verbal fluency (*p* = 0.04), which is also a reflection of frontal lobe dysfunction and by a trend for lower values in semantic verbal fluency (*p* = 0.06) (Figures 1C,D). Prominent impairment of verbal fluency in the MSA-C subtype which is not related to motor disability has been already described by Bürk et al. (28).

Interestingly, cortical thickness analysis revealed another clue for primary language dysfunction in MSA. There was a significant correlation between the baseline CERAD word list learning item and the left superior temporal gyrus thickness, as an important region for language processing. This is in high accordance with a recent meta-analysis of whole brain voxel-based morphometry in MSA, reporting cerebral cortex atrophy in both MSA subtypes, with predominant affection of the superior temporal gyrus (29).

Generally, there is no consensus regarding pattern difference comparing the MSA-C and the MSA-P motor subtype due to conflicting results (4). One study reported no difference in cognitive performance between both subtypes (30) whereas there are also reports on a more prominent impairment of memory and executive functions in MSA-C (31). In our current trial, in addition to the differences in language function, we also report a poor performance of patients with MSA-C in the TMT A and B tasks (Figures 1E,F) but not in the TMT B/A ratio compared to MSA-P. As already mentioned above, ratios have been described to reduce the impact of individual variability and to remove the components of motor speed and visual scanning speed from the TMT-B item (32). Therefore, we suspect that poor performance in TMT-A and B in the absence of worsening of the TMT B/A ratio might be mainly due to more severe motor impairment in the patients with MSA-C due to the cerebellar dysfunction, but we cannot exclude poor executive function as a contributing component.

A total of 14/20 patients in our cohort had longitudinal follow-up assessments (25–75p: 13–45 months). As a limitation of this study, due to patient dropout, we had to pool the patients with MSA-C and MSA-P to allow valid statistical evaluation of changes over time, applying multiple time points. Here, a significant worsening in TMT-B (*p* = 0.03) could be verified whereas all other CERAD items remained stable. Considering the TMT-A raw data, a trend for TMT-A worsening could be suspected but did not reach statistical significance. The more pronounced worsening of TMT-B compared to TMT-A cannot be solely attributed to influences by motor worsening despite the normal TMT B/A ratio. In contrast to TMT A, the TMT B item is a more complex task and also involves cognitive set shifting (33). Therefore, TMT-B worsening in this instance hints to the progression of frontal-executive dysfunction.

We cannot completely exclude that some patients not only dropped out because of progressive motor disability but also due to progressive cognitive dysfunction affecting cognitive domains others than the frontal execution. Additionally, higher patient number might have revealed statistically significant differences in other domains. Nevertheless, our report on executive dysfunction as the hallmark of cognitive impairment in MSA is in accordance with the literature (4).

Only few longitudinal studies can be found on the evolution of cognitive impairment in MSA and available studies mainly applied no more than one follow-up assessment after the initial baseline visit (8–13). Therefore, comparison of our current study including multiple time points of follow-up is only possible to a limited extent. Nevertheless, worsening of executive functions over time has been reported most frequently (11–13).

Generally, the pattern of prominent executive dysfunction in MSA shows a big overlap with other alpha-synucleinopathies and may not be used as a sensitive marker for differential diagnosis. Nevertheless, some factors might be helpful: fluctuation of cognition presents a core feature of Dementia with Lewy bodies (DLB) (34). Even though slight fluctuations (e.g., in association with orthostatic dysfunction) cannot be excluded in MSA, prominent DLB-like fluctuations are not common in MSA. Additionally, occurrence of visual hallucinations has been reported in 73% of patients with DLB and in 50% of patients with PD, whereas in MSA, visual hallucination occurred in <10% (35).

In conclusion, we give further evidence for frontal-executive dysfunction as the major characteristic of cognitive impairment in MSA also presenting the cognitive domain with the most prominent dynamics over time. Further multi-center studies including more patients and longer time periods are needed to characterize cognitive dysfunction and its evolution in MSA in greater detail which will be fundamental for the implementation into the diagnostic criteria on MCI/dementia in MSA.

## Data Availability Statement

The raw data supporting the conclusions of this article will be made available by the authors, without undue reservation.

## Ethics Statement

The studies involving human participants were reviewed and approved by Ethics Committee of the University Medical Center Göttingen. The patients/participants provided their written informed consent to participate in this study.

## Author Contributions

FM, PH, DV, SN, CR, AJ, NF, MH, MB, and IZ: design, conceptualization, and execution of the study. FM, IZ, and AL: execution and interpretation of the biostatistical analysis. FM, AJ, NF, and MH: execution and interpretation of the MRI/cortical thickness analysis. FM and IZ: drafting the manuscript. FM, PH, DV, SN, CR, AJ, NF, MH, MB, AL, and IZ: revising the manuscript. All authors read and approved the final manuscript.

## Conflict of Interest

The authors declare that the research was conducted in the absence of any commercial or financial relationships that could be construed as a potential conflict of interest.

## Publisher's Note

All claims expressed in this article are solely those of the authors and do not necessarily represent those of their affiliated organizations, or those of the publisher, the editors and the reviewers. Any product that may be evaluated in this article, or claim that may be made by its manufacturer, is not guaranteed or endorsed by the publisher.
